# **Graphene oxide and fluorescent aptamer based novel biosensor for detection of 25-hydroxyvitamin D**_3_

**DOI:** 10.1038/s41598-021-02837-4

**Published:** 2021-12-06

**Authors:** Ritika Gupta, Sunaina Kaul, Vishal Singh, Sandeep Kumar, Nitin Kumar Singhal

**Affiliations:** 1 Food and Nutritional Biotechnology, National Agri-Food Biotechnology Institute (NABI), Sector-81, S.A.S. Nagar, Mohali, Punjab India; 2 Department of Bio and Nano Technology, Guru Jambheshwar University of Science and Technology, Hisar, Haryana 125001 India

**Keywords:** Imaging and sensing, Fluorescence resonance energy transfer, Fluorescence spectroscopy

## Abstract

For maintaining the healthy metabolic status, vitamin D is a beneficial metabolite stored majorly in its pre-activated form, 25-hydroxyvitamin D_3_ (25(OH)D_3_). Due to its important role in bone strengthening, the study was planned to quantify 25(OH)D_3_ levels in our blood. Quantification techniques for 25(OH)D_3_ are costly thus requiring a need for a low cost, and sensitive detection methods. In this work, an economic, and sensitive sensor for the detection of 25(OH)D_3_ was developed using aptamer and graphene oxide (GO). Aptamer is an oligonucleotide, sensitive towards its target, whereas, GO with 2D nanosheets provides excellent quenching surface. Aptamer labeled with fluorescein (5’, 6-FAM) is adsorbed by *π–π* interaction on the GO sheets leading to quenching of the fluorescence due to Förster resonance energy transfer (FRET). However, in the presence of 25(OH)D_3_, a major portion of aptamer fluorescence remains unaltered, due to its association with 25(OH)D_3_. However, in the absence, aptamer fluorescence gets fully quenched. Fluorescence intensity quenching was monitored using fluorescence spectrophotometer and agarose gel based system. The limit of detection of 25(OH)D_3_ by this method was found to be 0.15 µg/mL whereas when GO-COOH was used, limit of detection was improved to 0.075 µg/mL. Therefore, this method could come up as a new sensing method in the field of vitamin D detection.

## Introduction

Vitamin D (Vit D) is a major regulatory fat soluble secosteroid moiety governing the skeletal growth and calcium homeostasis. Cholecaciferol (Vit-D_3_/25(OH)D_3_) being the stable form of Vit-D is the measurable moiety in serum. Low levels of Vit-D have been associated with health conditions like Osteomalacia, Depression, Parkinson’s disease, Autoimmune diseases and Cancer^1,2^. Thus, testing of Vit-D levels can be considered as an essential marker to estimate the quality of life of a population. Serum values lower than 75 nmol/L or 30 ng/ml are considered to be Vit-D deficient^3^. According to latest reports, Vit-D deficiency is found prevalent in developing and developed countries alike. With 5.9% population in US, 7.4% in Canada, 13% in Europe and > 20% population in countries like India, Pakistan and Afghanistan. 490 million people have been estimated to be Vit-D deficient in India^4^. Thus, the levels being on lower side, mass testing is required for the well-being of the population. Currently techniques used for the serum estimations are radioimmunoassay and chemiluminescent immunoassay. Other techniques available are High Performance Liquid Chromatography (HPLC–UV) and liquid chromatography combined with Mass spectroscopy (LC–MS). Chromatographic techniques have an add on feature of separate detection for 25(OH)D_2_ and 25(OH)D_3_^5^. Though the detection of 25(OH)D_3_ with the presently used methods is possible but have limitations of their own. Immunoassays having poor antibody specificity with issues of cross-reactivity while chromatography techniques involving costly instruments that require trained staff for operation^6,7^. Due to these cost limitations and high maintenance issues, status monitoring of 25(OH)D_3_ is hampered. Thus, there is a need to have point of care instrumentation for testing^8^.

To overcome these issues, scientists have been successful in developing electrochemical detection systems. Immunosensor reported by Kaur et al., provided a quick response time of 12 min and stability of 10 days. Bimetallic Au-Pt nanoparticles were synthesized that were later deposited on APTES modified electrode. Immobilization of Ab-25(OH)D_3_ played the role of providing specificity and sensitivity to the electrode^9^. On similar grounds, Anusha et al., developed a glassy carbon electrode surface for immobilization of Lanthanum nanoparticles-graphene quantum dots coupled with zeolitic imidazolate framework (ZIF-8). Sensitive detection of Vit-D in human serum and urine samples was made possible by this method^10^. Chauhan et al., have been working on different procedures for Vit-D detection and have come up with electrochemical sensors using polyacrylonitrile nanofibers with incorporated magnetic nanoparticles and gadolinium oxide nanorods. They were able to obtain enhanced sensitivity of metal oxide nanorods as compared to nanofibers^11^. On the contrary, Lee et al., developed a gold nanoparticle based platform for colorimetric quantification of Vit-D levels in serum samples on smartphone^12^.

Recently, aptamers have successfully come up as a detection tool that is stable, cheap and reproducible^13^. They are oligonucleotide equivalent of antibodies having specificity towards particular metabolites^14^. They generally adopt secondary structures like stem, loop, G-quadruplex and hairpin that further form 3D conformations in presence of particular metabolites which is responsible for their binding affinity and specificity^15–17^. SELEX (systematic evolution of ligands by exponential enrichment) is the technique that allows the aptamers to segregate depending on their specificity towards their target moieties^18^. Forces enhancing the aptamer target binding involve non-covalent interactions including electrostatic interactions, hydrogen bonding, Van der Waal’s interaction and shape complementarity^19^. In order to be used as sensors, aptamers can be labeled with fluorophores, gold nanoparticles and quantum dots that can be visualized on their respective binding^20–22^.

Graphene oxide (GO), one atom thick sheet of oxidized graphite has been efficiently used as fluorescence quencher^23^. Due to its solubility, inertness and surface functionality, GO has been a part of many detection systems^24,25^. It acts as an excellent quencher of fluorescence by the process of FRET. FRET (Förster resonance energy transfer) is the phenomenon of energy exchange between molecules associated in < 10 nm of range^26^. Fluorophore tagged to the hairpin aptamer loses its fluorescence when in contact with GO due to π stacking interactions between the nucleobases of ssDNA and π ring of GO. Utilizing the specificity benefits of FRET based assay, sufficient work has been published on detection of biomolecules such as thrombin^27^, pfldh^20^, adenosine^28^, kanamycin^29^. There is a lack of Vit-D based detection systems utilizing the similar phenomenon. Considering the stability, specificity and low cost of aptamers we planned to prepare a FRET based assay for Vit-D detection with the help of above explained VDBA-14 aptamer.

**Figure 1:**
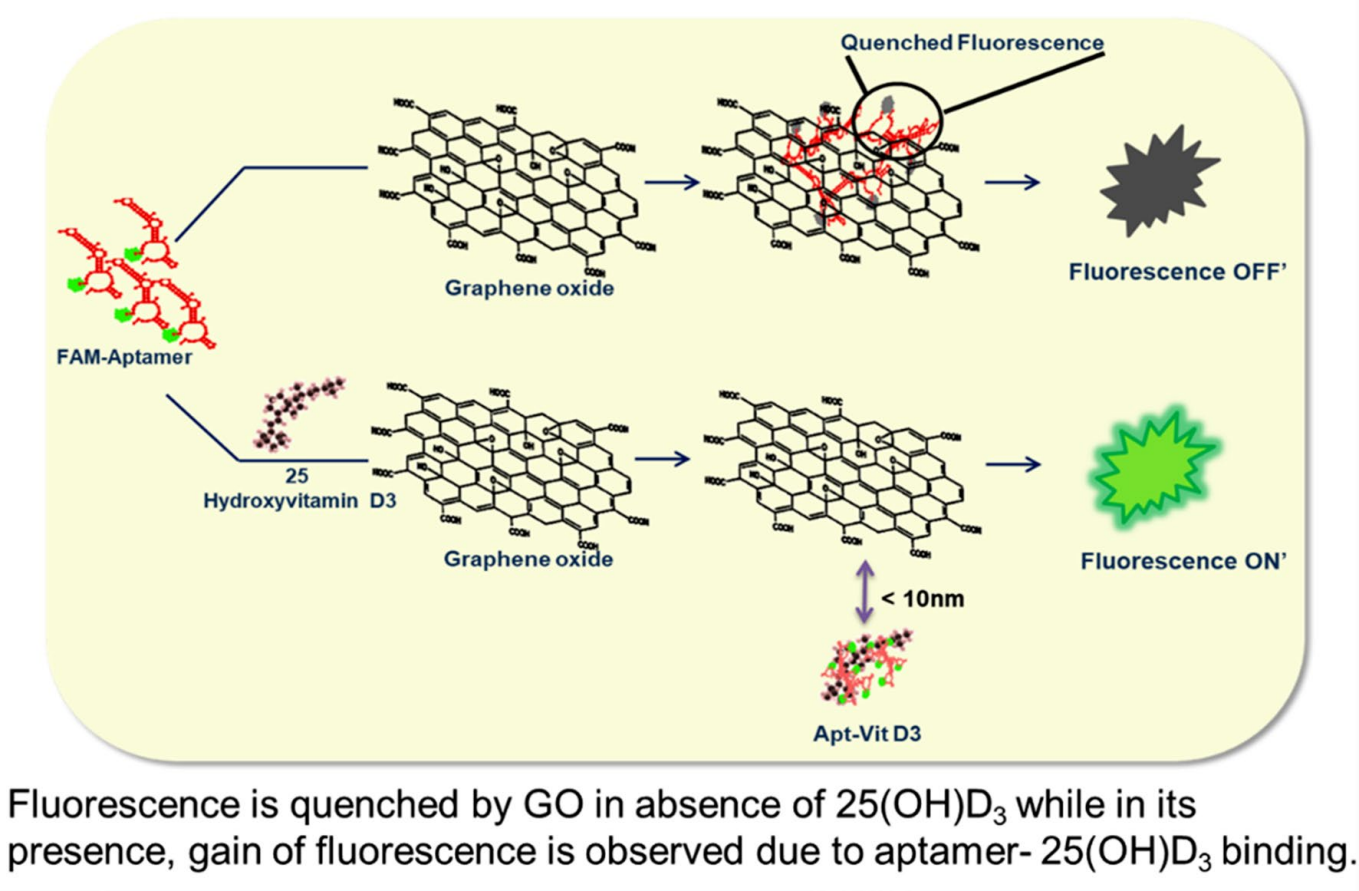
Schematic representation of FAM-Aptamer. Fluorescence is quenched by GO in absence of 25(OH)D_3_ while in its presence, gain of fluorescence is observed due to aptamer-25(OH)D_3_ binding.

In this study, a fluorescent tagged aptamer based detection system was developed for the identification of 25(OH)D_3_. Hairpin loop aptamer, stacks on GO due to its π–π and hydrophobic weak interactions causing the associated quenching (Fig. 1). As already mentioned quenching occurs due to FRET phenomenon^30^. Conversely in the presence of 25(OH)D_3_, aptamer having high affinity towards its metabolite aligns its conformation around it, leaving the vicinity of GO, increasing the distance between fluorophore and GO thus, producing fluorescence. 25(OH)D_3_ detection utilizing the FRET^31^ phenomenon and the gel based detection have not been studied so far, thus producing the novelty factor of the work. The above phenomenon was also validated by spectral changes and as well as agarose gel electrophoresis.

## Results/discussions

### Characterization of GO

**Figure 2:**
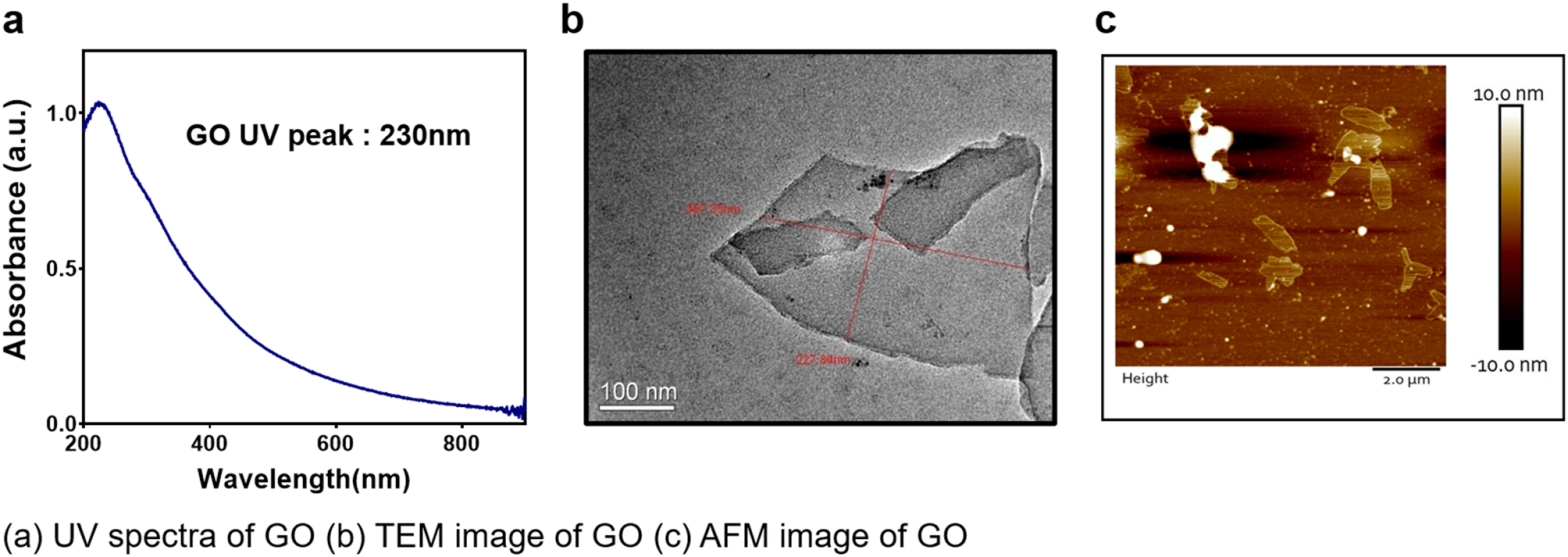
Characterizations of synthesized Graphene Oxide. (**a**) UV spectra of GO (**b**) TEM image of GO (**c**) AFM image of GO.

Ultraviolet visible spectroscopy (Shimadzu, UV-2700) and Transmission electron microscopy (JEOL, JEM 2100, operating at 200 kV) were used for the characterization of prepared GO. In Fig. 2a GO absorbs at 230 nm which attribute to the π–π* transition of aromatic C–C bond and also represent π–π* plasmon peak. TEM was carried out to see the surface morphology which clearly demonstrates the single layer structure of GO and the same can be seen in Fig. 2b. thickness of which can be estimated by Atomic Force Microscopy (AFM) results (Fig. 2c). Thickness of the exfoliated GO sheets were estimated to be < 5 nm by AFM (Bruker 8 multimode scanning microscope). Carboxylated Graphene oxide was characterized using UV–Vis spectroscopy, shoulder peak n–π* of Graphene oxide at 300 nm was removed after carboxylation. Since the hydroxyl group has converted to carboxyl (COOH) group, negative charge has been increased from − 43 to − 53 and also the COOH peak in FTIR has been increased (Fig.S1).

### Optimization of graphene oxide concentration for efficient quenching

**Figure 3:**
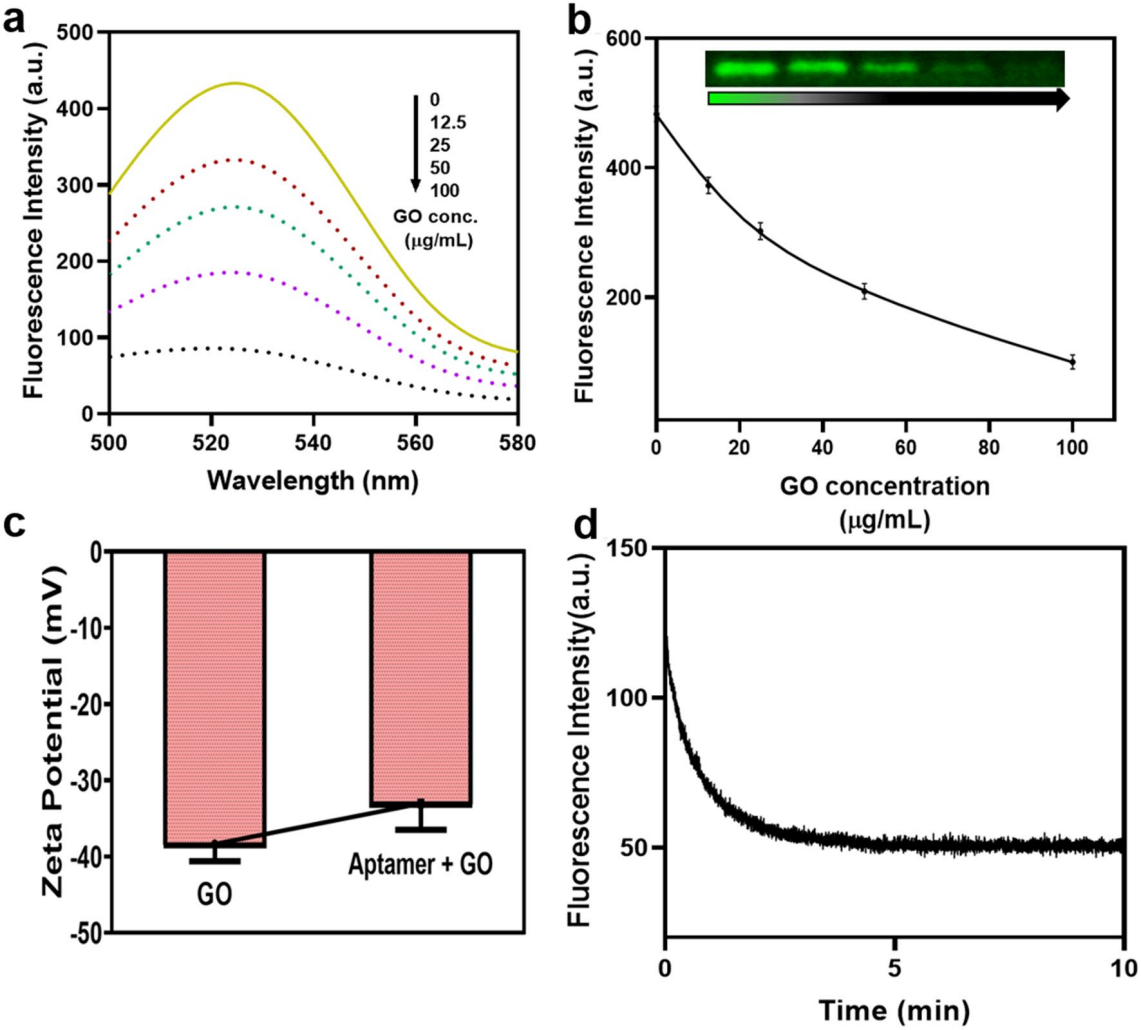
Graphene oxide and aptamer optimization. (**a**) Fluorescent spectra of aptamer with various concentration of GO. (12.5 µg/mL, 25 µg/mL, 50 µg/mL and 100 µg/mL) (**b**) Quantitative and qualitative (agarose gel) results of aptamer fluorescence with increasing concentration of GO. Inset: Peak fluorescence change shows a linear relationship with Graphene oxide concentration. (**c**) Zeta potential of Apt and Apt-GO (**d**) Time kinetics of fluorescence quenching in presence of 50 µg/mL.

Aptamer used in the study is β-hairpin loop ssDNA. Sequence and structure (Fig.S2) were given in supplementary information. GO has come up as an effective energy acceptor due to its unique electronic properties^29^. Thus, the strategy used for developing the assay involved fluorescence quenching of the FAM-aptamer in the presence of GO. FAM modified aptamer when present in vicinity of GO was able to get its fluorescence quenched due to the phenomenon of FRET. In the presence of Vit-D_3_, affinity of aptamer towards Vit-D allowed it to majorly bind to the target molecule leaving few unbound aptamers to be adsorbed on GO sheets by π stacking interactions. This assay allowed enhanced fluorescence values with respect to increasing concentrations of Vit-D. The amount of quenching obtained was observed by allowing the FAM-aptamer to be mixed with GO followed by centrifugation. Supernatant was used for the spectral studies as shown in Fig. 3a. Quenching of fluorophore was proportional with increasing concentration of GO. As given in Fig. 3b, we deduced that more than 60% quenching was observed when 50 µg/mL GO was used^31,32^. Thus, 50 µg/mL was selected as the optimum concentration for the quenching studies. Fluorescence intensity was taken using excitation at 495 nm and emission from 500 to 580 nm on Agilent 121 Cary Eclipse Fluorescence Spectrophotometer. Qualitative analysis of fluorescence quenching can also be visualized on 3% agarose gel electrophoresis, image was taken on Azure Biosystems c600 [whole gel image given in Fig.S7(a)]. The zeta potential taken on Malvern, Zetasizer, Nano-ZS shown in Fig. 3c, decreased from − 38 mV to − 33 mV when GO non-covalently interacted with FAM-apt. Thus, indicating capping of a few negative charges of GO by nucleobases of ssDNA FAM-aptamer. Figure 3d represent the time required for sufficient quenching of fluorescence in the presence of GO. Minimum 5 min were required for maximum quenching. Thermogravimetric analysis (TGA) was performed on PerkinElmer TGA 8000. As could be visualized from Fig S3(a), fall in weight% upto 100 °C corresponded to loss in adsorbed water. Degradative step commencing at 165 °C was indicative of loss of hydroxyl and epoxy functional groups. The second fall in weight from 400 °C was indicative of remaining oxygen containing groups^33^. Difference of 2% weight loss was obtained in GO-Apt till temperature 100 °C and from 200 °C to 600 °C. Functional groups characteristic for GO were analyzed through FTIR PerkinElmer UATR Two instrument. FTIR spectra clearly shows peaks at 1591.6 cm^−1^, 1374.41 cm^−1^ and 1075.99 cm^−1^ indicative of C=C, O–H and C–O–C respectively as can be observed from Fig S3(b) ^34,35^. On addition of aptamer, significant changes were not observed due to weak π–π interactions between aptamer and GO. Fig. S3(c) showed characteristic peak of GO at 230 nm that corresponded to π–π* transition of C=C bonds^35^. Peak of aptamer was seen at 260 nm. Slight shift in spectra could be observed in GO-Apt. To improve the quenching efficiency, GO-COOH was used and found that the quenching was increased by approximately 15% at 50 µg/mL. Initially, quenching with 50 µg/mL GO was 65% but with GO-COOH, it was increased to more than 80% Fig S4. The result was similar to previous study done where GO quenching was compared with functionalized GO-COOH^36^.

### Condition optimization and 25-hydroxyvitamin D_3_ detection

For pH optimization, aptamer and 25(OH)D_3_ were added in different pH buffer (3.5, 5.5, 7.4, 8.3, and 9.2). As acidic pH caused charge neutralization on the GO surface, (H^+^ charge abundance nullified the negative charges present on GO), the electrostatic forces between aptamer and GO were enhanced and caused complete quenching of the aptamer the π-π stacking forces between aptamer and GO were enhanced and caused complete quenching of the aptamer even in the presence of 25(OH)D_3_^37^. The interactions were so strong that even the presence of 25(OH)D_3_ was insufficient to regain the fluorescence. Fig S5(a), maximum fluorescence was detected in pH 7.4 and 8.3 reaction mixtures. At pH 9.2, as reaction mixture became alkaline, negative charge accumulation caused repulsion between aptamer and GO. Thus, causing fluorescence to maintain and very less quenching was observed. Similar results were also confirmed from agarose gel electrophoresis (3% agarose gel in 1X TBE buffer, pH8.3) as shown in Fig. S5(a) and whole gel image Fig. S7(d). As can be observed from the relative fluorescence intensity values (F1/F0), F1 is the final fluorescence obtained after addition of 25(OH)D_3_ in reaction mix containing aptamer and graphene oxide, while F0 refers to the fluorescence of only FAM-aptamer. (For convenience in comprehending we have shown comparison with only quenched aptamer in the manuscript. The only aptamer data is included in supplementary file).

To obtain the optimum visualization, effect of temperature on aptamer and 25(OH)D_3_ (5 µg/mL) binding was also studied. Aptamer and 25(OH)D_3_ were incubated at different temperatures (4 °C, 16 °C, 25 °C, 37 °C and 50 °C). It was concluded from Fig S5(b) that 37 °C was appropriate for efficient aptamer and 25(OH)D_3_ binding. Agarose gel showing fluorescent bands confirms the 37 °C is the optimum temperature for the assay with complete gel image in Fig. S7(e).

**Figure 4:**
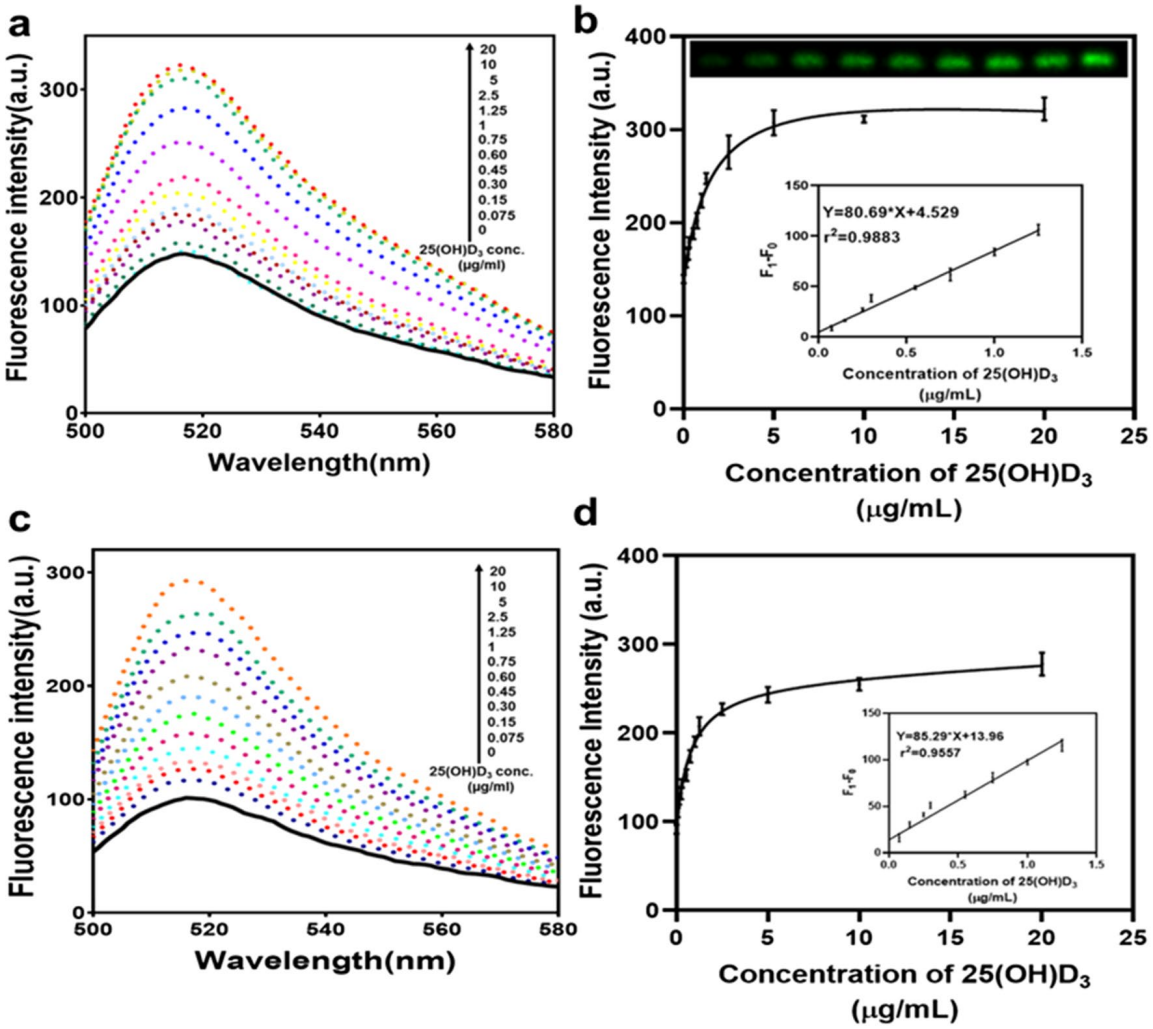
Assessment of fluorescence intensity obtained with 25(OH)D_3_. (**a**,**b**) Fluorescent spectra and fluorescent intensity of the aptamer with different conc. of 25(OH)D_3_ and 3% agarose gel image confirming the increase in fluorescence in presence of 25(OH)D_3_ using GO. Inset: Peak fluorescence change shows a linear relationship with 25(OH)D_3_ concentration (0–1.25 µg/mL). (**c**,**d**) Fluorescent spectra and fluorescent intensity of the aptamer with different conc. of 25(OH)D_3_ using GO-COOH. Inset: Peak fluorescence change shows a linear relationship with 25(OH)D_3_ concentration (0–1.25 µg/mL). (Apt –100 nM, GO and GO-COOH-50 µg/mL) F0: Aptamer + GO (**b**) and Aptamer + GO-COOH (**d**); and F1: Apt + GO + 25(OH)D_3_ (**b**) and Apt + GO-COOH + 25(OH)D_3_ (**d**).

Aptamer and different concentration of 25(OH)D_3_ were allowed to bind for 45 min with gentle mixing. After the incubation time was over, free aptamers got stacked on the GO surface. On centrifugation, GO bound aptamers settle in pellet while 25(OH)D_3_ bound aptamers remained in the supernatant that was detected through spectral absorption and also validated by 3% agarose gel. In Fig. 4a fluorescent intensity spectrum shows that with the increase in the 25(OH)D_3_ fluorescence increases. The same is confirmed by the relative fluorescence measure where in the presence of 25(OH)D_3_, fluorescence signal is observed. It is similar to the on/off kind of assay where the signal is on in the presence of the analyte and off in its absence. Minimum concentration of 25(OH)D_3_ that was detected using this assay was 0.30 µg/mL with precision but can detect 0.15 µg/mL as confirmed by Fig. 4b and Fig S7 (b). The linear range observed was 0.075 to 1.25 µg/mL and linear equation y = 80.69x + 4.527. Limit of detection using 3σ/m through linear equation was found to be 0.0537 µg/mL. We could also see the increase in FAM signal in the agarose gel which again proves the reliability of the assay. In case of GO-COOH, since the quenching was more than only GO, and aptamer was also able to release due to its affinity at low concentration of 25(OH)D_3_ therefore, it was observed that the probe with GO-COOH was able to detect 0.075 µg/mL thus improving the LOD of the assay as seen in Fig. 4c,d. The linear range observed was 0 to 1.25 µg/mL and linear equation y = 82.29x + 13.96. In order to compare the efficacy of the present study with already existing tools for 25(OH)D_3_ detection, Table 1. is given.

**Table 1:** Comparison of various techniques available for detection of Vit D.

Analytical method	Detecting moiety	Target	Limit of Detection	References
Radioimmunoassay	Antibody	Vit-D	2.8 ng/mL	^41^
Bimetallic nanoparticles on APTES modified glass electrode	Antibody	Vit-D	0.0049 ng/mL	^9^
Chemiluminiscence immunoassay	Antibody	25(OH)D	1.43 ng/mL	^38^
Aptasensor using Graphene Quantum Dot-Au hybrid nanoparticles	Aptamer	25(OH)D_3_	0.28 ng/mL / 0.70 nM	^42^
Electrochemical immunosensor	Antibody	Vit-D_3_	0.12 ng/mL	^11^
Electrochemical sensor using Gadolinium oxide nanorods	Antibody	Vit-D_3_	0.10 ng/mL	^43^
Enzyme modified electrode	Enzyme based redox reaction	25(OH)D_3_	5-200 ng/mL	^44^
Aggregation of gold nanoparticles producing colorimetric change	Aptamer	25(OH)D_3_	1 µM	^12^
FRET	Aptamer	25(OH)D_3_	75 ng/mL / 0.187 µM	Our work

### Specificity check and real-time sample analysis in mice serum

**Figure 5:**
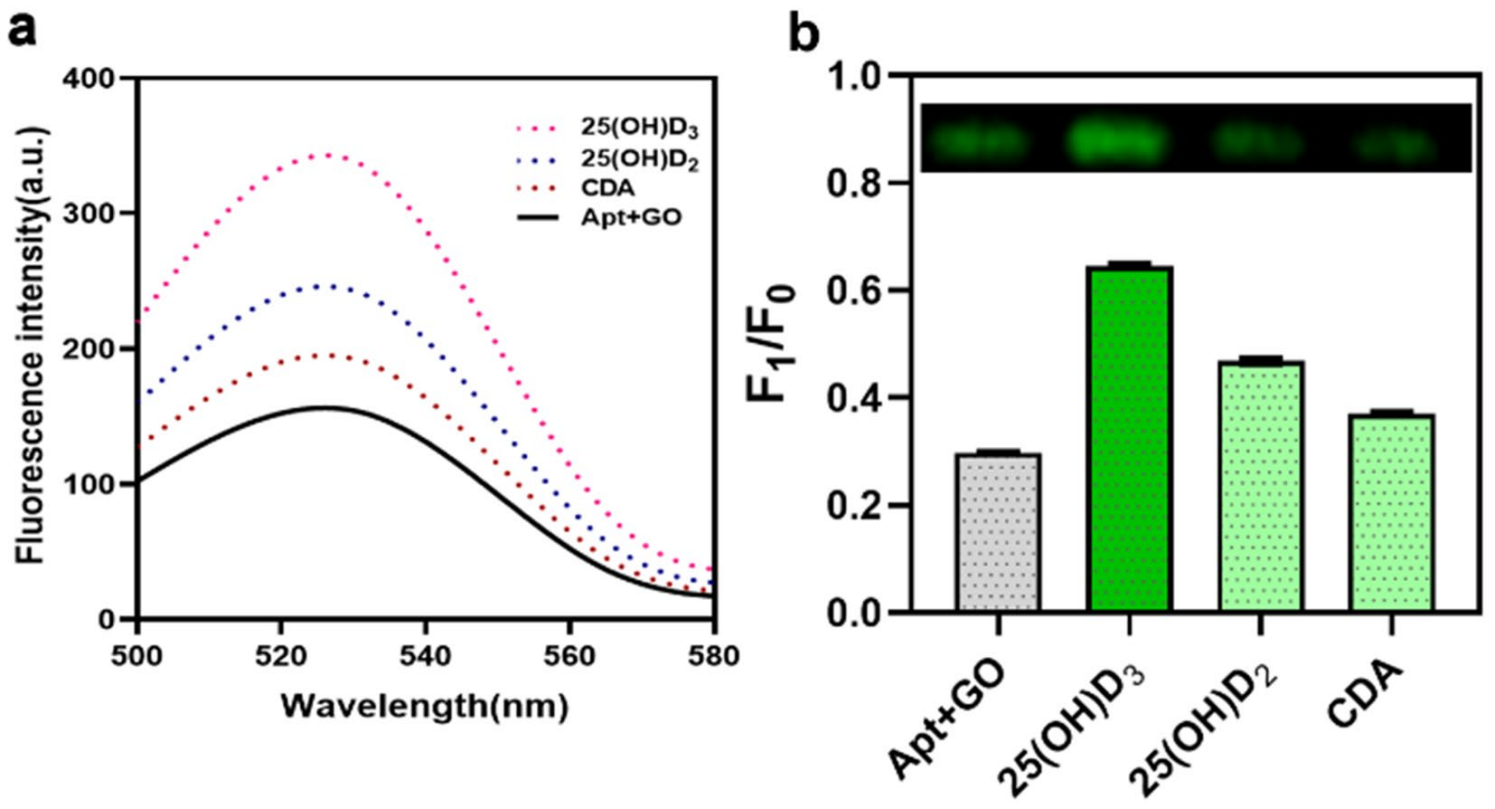
Specificity evaluation for 25(OH)D_3_ detection. (**a**) Aptamer binding specificity check with different negative controls 25(OH)D_2_ and CDA. Maximum fluorescence intensity was observed in 25(OH)D_3_ when compared to the other compounds proving the specificity of the aptamer. (**b**) Quantitative and qualitative analysis was also proved using 3% agarose gel electrophoresis. [Apt – 100 nM, GO-50 µg/mL and 25(OH)D_3_-5 µg/mL] F0: only Aptamer which is 1 and F1: Apt + GO in the presence and absence of compounds. The comparison of negatives was expressed as Mean ± S.D., n = 3.

Specificity reaction involved separate aliquots of aptamer to be allowed to bind to 25(OH)D_3_ similar moieties such as 25(OH)D_2_ and CDA in order to check for cross reactivity. Maximum fluorescence intensity was observed in 25(OH)D_3_ as can be seen in Fig. 5a,b as compared to the other compounds proving the specificity of the aptamer. Gel quantitative analysis also proves the specificity of the assay [Figs. 5b and S7c]. Experiment was also performed with two scrambled aptamers to prove the specificity of the binding of aptamer with target. It was observed that both the scrambled as well as the specific aptamer was quenched but regaining was observed only in case of specific 25(OH) D_3_ aptamer as can be seen in Fig S6. Hence, it can be said that the target specifically binds only to its specific aptamer.

**Figure 6:**
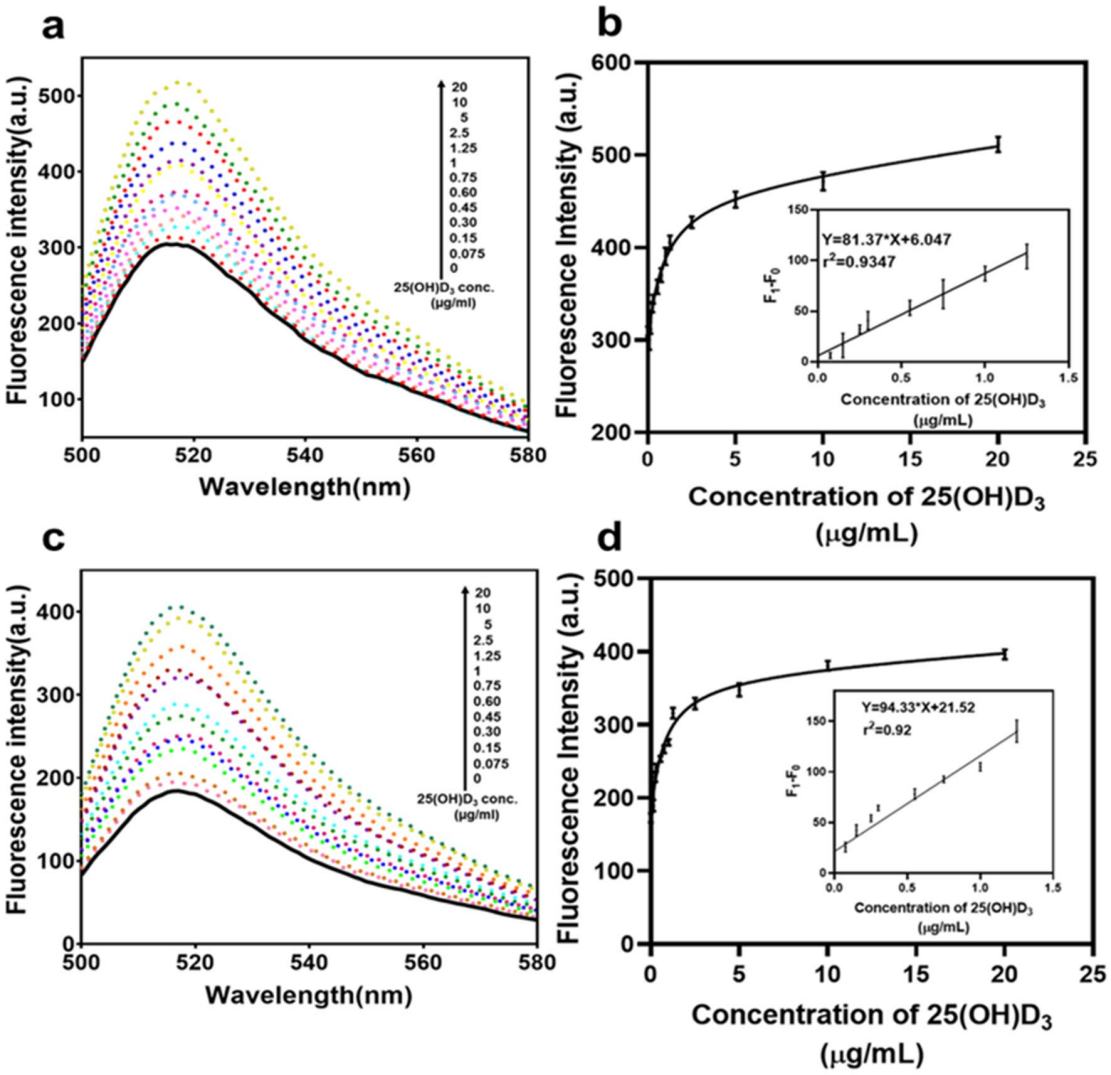
Evaluation for 25(OH)D_3_ detection in Mice serum. (**a**,**b**) Fluorescent spectrum and fluorescent intensity of real time samples analysis using 50% mice serum spiked up samples with 25(OH)D_3_ using GO Inset: Peak fluorescence change shows a linear relationship (0–1.25 µg/mL) with 25(OH)D_3_ concentration. (**c**,**d**) Fluorescent spectrum and fluorescent intensity of real time samples analysis using 50% mice serum spiked up samples with 25(OH)D_3_ using GO-COOH Inset: Peak fluorescence change shows a linear relationship (0–1.25 µg/mL) with 25(OH)D_3_ concentration. (Apt –100 nM, GO and GO-COOH-50 µg/mL) F0: Aptamer + GO (**b**) and Aptamer + GO-COOH (**d**); and F1: Apt + GO + 25(OH)D_3_ (**b**) and Apt + GO-COOH  + 25(OH)D_3_ (**d**).

25(OH)D_3_ was supposed to be detected in human serum but in order to prove the usefulness of the assay, we detected 25(OH)D_3_ in mice serum. Mice sera was used to mimic the human serum. In 50% serum, fluorescence is higher than in water or buffer^38^. In this case, sufficient quenching was observed in absence of 25(OH)D_3_ whereas in the presence of 25(OH)D_3_ spiked serum sample, fluorescence intensity was increased when compared with no 25(OH)D_3_ control as could be observed in Fig. 6a,b. Using carboxylated GO, the limit of detection in mice serum was also increased similar to the assay performed in buffer as observed in Fig. 6c,d.

## Materials and methods

### Materials

25-hydroxyvitaminD_3_ (25(OH)D_3_), 25-hydroxyvitaminD_2_ (25(OH)D_2_), Chenodeoxycholic acid (CDA), Tris, Sodium chloride, Sodium Hydroxide, Chloroacetic acid, Graphite flakes from Sigma (www.sigmaaldrich.com), Agarose from Lonza (www.lonza.com). Aptamer specific to vitamin D was taken from literature^12^ and synthesized with 6-carboxyfluorescein (FAM) modifications at its 5’end by Sigma Aldrich (technical datasheet given in supplementary information). 96 well black round bottom plate from Corning, Thermoscientific (www.thermoscientific.com). Mice serum was taken from animal house facility at National Agri-Food Biotechnology Institute, Mohali, India under the ethical no. NABI/2039/CPCSEA/IAEC/2019/13. The blood was withdrawn from retro-orbital plexus of the mice and serum was isolated and utilized for the further experiments.

### Synthesis and characterizations of graphene oxide (GO)

GO was prepared by modified Hummers’ method^39^. Briefly, a 9:1 mixture of concentrated H_2_SO_4_/H_3_PO_4_ (360:40 mL) was added to a mixture of graphite flakes (3.0 g, 1 wt. equiv.) and KMnO_4_ (18.0 g, 6 wt. equiv.). The reaction was then heated to 50 °C and stirred for 12 h. The reaction was cooled to RT and poured onto ice (400 mL) with 30% H_2_O_2_ (3 mL). Then filtered through muslin cloth. The filtrate was centrifuged (5380*g* for 30 min), and supernatant decanted. The remaining solid material was then washed two times in succession with 200 mL of water, 200 mL, 30% HCl, and 200 mL, ethanol and after each wash, the mixture was filtered through muslin cloth with the filtrate being centrifuged (5380*g* for 30 min) and the supernatant decanted away. The remaining material after washing was coagulated with 200 mL of ether, and centrifuged at 5380*g* for 30 min to remove ether. The pellet obtained was dissolved in water and 0.1 M NaOH was added to make homogenous suspension followed by dialysis using deionized water and lastly lyophilized overnight at room temperature to find the concentration of the obtained GO. Prepared graphene oxide was characterized by UV–Vis spectroscopy, TEM imaging and AFM analysis. Carboxylated Graphene oxide was prepared as per the protocol mentioned in literature^40^. Briefly, 30 ml of Graphene oxide (2 mg/mL) was sonicated for 1 h followed by addition of 7.2 g of NaOH (sodium hydroxide) and 6 g of Cl–CH2–COOH (Chloroacetic acid) and sonicated for 3 h. The formed suspension was washed thrice at 5380 g for 20 min to remove excess of NaOH and Cl–CH2–COOH. The formed carboxylated graphene oxide (GO-COOH) was characterized by UV–vis spectroscopy, dynamic light scattering and FTIR.

### Optimization of graphene oxide concentration, temperature and pH for efficient quenching and regaining assays

Aptamer (stock 1 µM) was prepared in 20 mM phosphate buffer, 25 mM NaCl, pH 7.4. To estimate the optimum concentration of GO, its varying concentrations (0, 12.5, 25, 50, 100 µg/ml) were incubated with aptamer, 100 nM (working concentration) for 10 min in 250 µl reaction mixture at room temperature. Centrifugation was followed at 18800*g* for 20 min. Supernatant obtained was separated, used for fluorescence estimations and for visualized confirmation on agarose gel 3% agarose in 1X TBE buffer (at 80 mV) with same concentration as used in fluorescence spectrophotometry assay. Similar experiment was performed with GO-COOH to observe the effect of carboxylation on quenching.

To study the effects of pH on the binding, 5 µg/mL of 25(OH)D_3_ was incubated with aptamer in varying reaction buffers, 20 mM sodium acetate buffer (pH-3.5, 5.5), 20 mM phosphate buffer (pH-7.4), 20 mM tris buffer (pH-8.3, 9.2). In order to find the optimum temperature, reaction mixture containing aptamer and 25(OH)D_3_, was incubated at 4 °C, 16 °C, 25 °C, 37 °C and 50 °C. After the appropriate condition for standardization was completed over 45 min, GO (optimized as above, 50 µg/ml) was added to the reaction mixture followed by centrifugation and later estimations. Similarly, experiment for pH and temperature was also performed on agarose gel using 3% agarose in 1X TBE buffer (at 80 mV). Samples were same as used in fluorescence spectrophotometry experiment.

### Fluorescence detection of vitamin D_3_

In a 250 µL reaction mixture, aptamer (100 nM) and 25(OH)D_3_ (0.075, 0.15, 0.3,0.45, 0.6,0.75 1.25, 2.5, 5, 10 and 20 µg/mL) were incubated in 20 mM phosphate buffer (25 mM NaCl, pH7.4) for 45 min with gentle mixing at 450 rpm in thermomixer (Eppendorf). GO (50 µg/mL) and GO-COOH (50 µg/mL) was added and allowed to mix for 10 min at room temperature. In control experiment, only aptamer and GO were taken without 25(OH)D_3_. Centrifugation was followed with supernatant removal without disturbing the pellet and analysis by fluorescence estimation and agarose gel based visualization (3% in 1X TBE buffer at 80 mV with same concentration as used in fluorescence spectrophotometry assay).

### Selectivity and real-time sample analysis

Closely resembling analytes, 25(OH)D_3_, 25(OH)D_2_ and CDA was used for the specificity check of the assay. 5 µg/mL of each biomolecule was diluted in 20 mM phosphate buffer, pH 7.4. Optimized concentrations of aptamer and GO was added and analyzed using the same procedure as given above for the detection of 25(OH)D_3_. To further prove the specificity of the probe, two scrambled aptamers (100 nM) were taken, incubated for 5 min with GO and 25(OH)D_3_, 5 µg/ml was added. Further experiment was followed as done previously. Experiment was also performed on agarose gel using 3% agarose in 1X TBE buffer (at 80 mV). Samples and sample concentrations were same as used in fluorescence spectrophotometry experiment.

In order to determine practical applications in human serum sample, mice serum was taken as a real-time sample. Serum was diluted to 50% with phosphate buffer, spiked up with 25(OH)D_3_ in dilutions ranging from 0.075, 0.15, 0.3,0.45, 0.6, 0.75 1.25, 2.5, 5, 10 and 20 µg/mL were used for the estimations. For the control experiment, non-spiked sample of 50% mice serum was used. Rest of the assay procedure was followed as described above using GO and GO-COOH.

### Statistical analysis

Results obtained were expressed as Mean ± S.D with n = 3 using Graph Pad Prism software for windows (version8).

## Conclusion

Vitamin D_3_ being an essential component of the body requires proper detection system. As already shown that using aptamer based detection techniques, quantification becomes specific without any cross reactivity, which makes it a sensitive assay using fluorescence techniques. Here, for the first time FRET assay was used for fluorescent detection of 25(OH)D_3_ and also for the visualization of the same on agarose gel. Increasing gradation of 25(OH)D_3_ caused fluorescence restoration in phosphate buffer as well as similar results was observed in even 50% serum. Even the assay utilizing VDBA 14 aptamer has been reported to have LOD of 1 µM which is far away from 0.006 µM as reported by Anusha et al.^8^. Improvement in this protocol by either increasing the specificity of the aptamer and reducing the fluorescence constraints can help in development of chip model and used as point of care testing device. Limit of detection using GO was 0.15 µg/mL with linear range (0.075 to 1.25 µg/mL). Limit of detection was improved to 0.075 µg/mL while using GO-COOH with linear range (0.075 to 1.25 µg/mL). From the results, we can see that the quenching and fluorescence restoration could be visualized through optical gel imaging. Thus, making optical detection also possible. This technique could be used on chip model and can be used as point of care testing device. Further modifications could make the method more sensitive in the future.

## Supplementary Information

Supplementary Information.
