# Supplementary material for: Graphene oxide and fluorescent aptamer based novel biosensor for detection of 25-hydroxyvitamin D3
Source: Sci Rep. 2021 Dec 6;11:23456. doi: 10.1038/s41598-021-02837-4 (PMC8649066; doi:10.1038/s41598-021-02837-4)

**Supplementary information**

**Graphene Oxide and Fluorescent aptamer based novel biosensor for detection of 25-hydroxyvitamin D_3_**

Ritika gupta^1ζ^, Sunaina kaul^1ζ^, Vishal Singh, Sandeep Kumar^2^, Nitin Kumar Singhal^1^*

^1^Food and Nutritional Biotechnology, National Agri-Food Biotechnology Institute (NABI), Sector-81, S.A.S. Nagar, Mohali, Punjab, India

^2^Department of Bio and Nano Technology, Guru Jambheshwar University of Science and Technology, Hisar, Haryana, 125001, India

**Figure S1. Characterization of GO-COOH in comparison to GO**

(a) UV-Vis spectroscopy (b) Zeta potential and (c) FTIR of GO and GO-COOH

**Figure S2. Sequence and structure of aptamer**

**Figure S3. Graphene oxide and aptamer characterizations**

(a) TGA of GO and GO-Apt (b) FTIR spectra of GO and GO-Apt (c) Combined UV spectra of GO, Apt and GO-Apt. (Apt – 100 nM, GO-50 µg/mL)

**Figure S4: Carboxylated GO (GO-COOH) concentration optimization**

(a) Fluorescence spectra of aptamer with different GO-COOH concentration (12.5µg/ml, 25 µg/ml, 50 µg/ml and 100 µg/ml) (b) Line graphs showing decrease in fluorescence with increase in GO-COOH concentration

**Figure S5: Optimization of pH and Temperature for aptamer and 25(OH)D_3_ binding**

(a) Binding of apt with 25(OH)D_3_ at different pH followed by addition of GO and agarose gel image confirming the binding of apt (b) Binding of apt with 25(OH)D_3_ at different temperature followed by addition of GO and agarose gel image showing the same. [In (a) * represent only aptamer, # - Apt + GO and § - Apt + 25(OH)D_3_ + GO]. (Apt – 100 nM, GO-50 µg/mL and 25(OH)D_3_- 5µg/mL for both the assays) F0: Only Aptamer and F1: Apt+GO in the presence and absence of 25(OH)D_3_.

**Figure S6: Specificity analysis with scrambled aptamers**

**Figure S7:** **Whole gel images**

Agarose gel results of (a) increasing concentrations of GO (Fig.3b) (b) increasing concentrations of 25(OH)D_3_ (Fig.4b) (c) negative controls (Fig.5b) (d) Effect of pH (Fig.S5a) (e) effect of temperature (Fig.S5b).

**Figure S8: Technical details of aptamers**

1. Characterization of carboxylated graphene oxide (GO-COOH) compared to graphene oxide (GO)


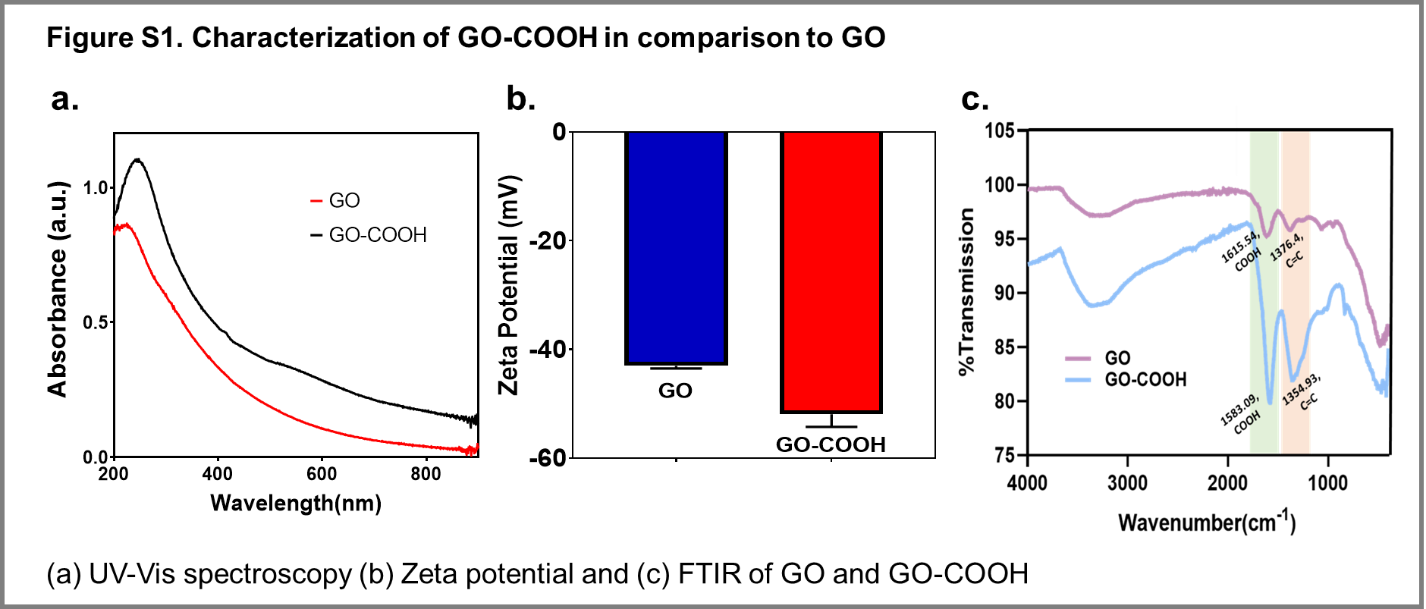


*2. Aptamer sequence of the selected sequence:*

AGCAGCACAGAGGTCATGGGGGGTGTGACTTTGGTGTGCCTATGCGTGCTACGGAA


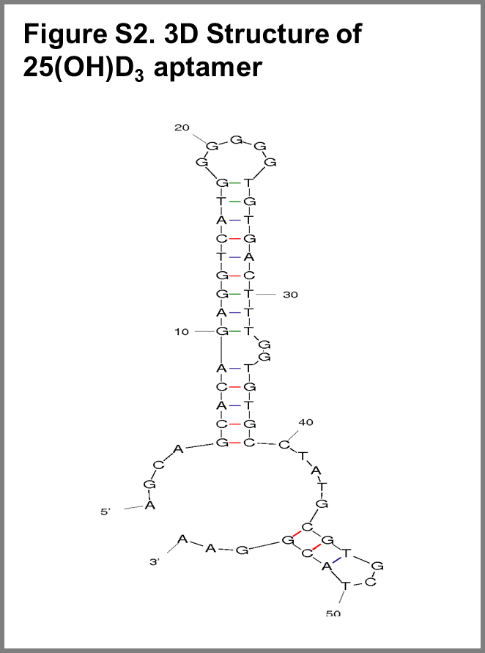


*3. Characterization of GO and GO-Apt*

*
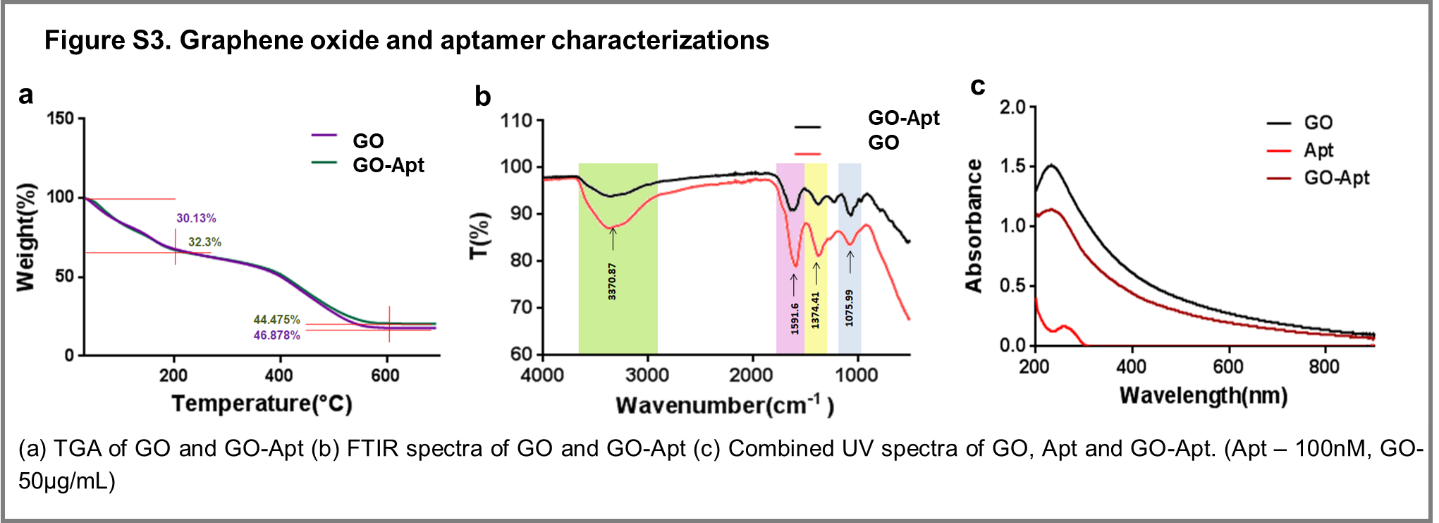
*

*4. Concentration optimization of Carboxylated graphene oxide*

*
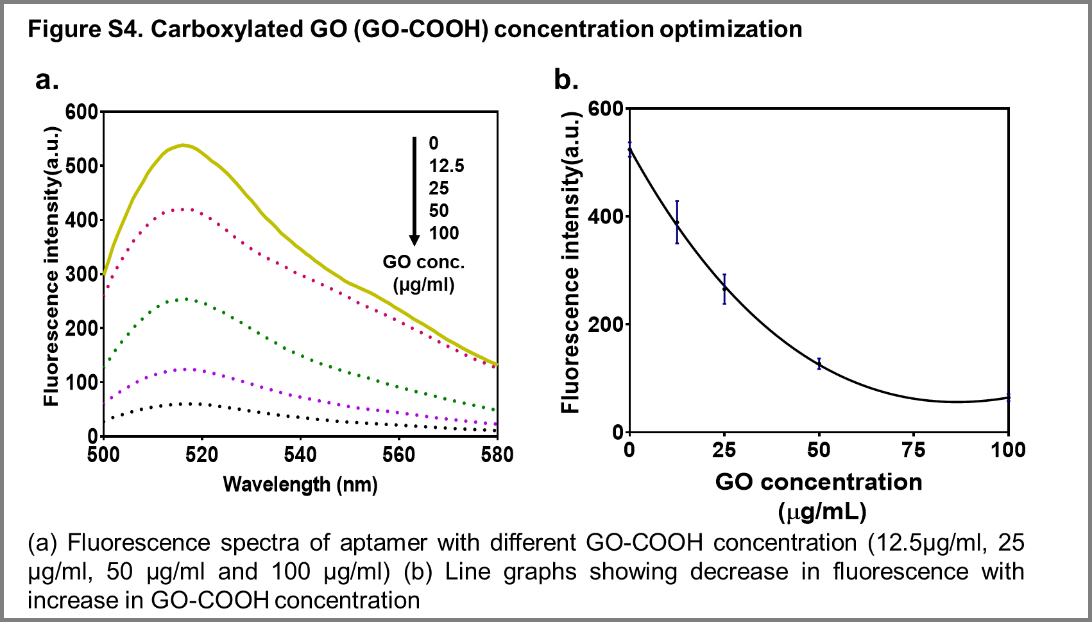
*

*5. Optimization of pH and Temperature for aptamer and Vit D_3_ binding*

**
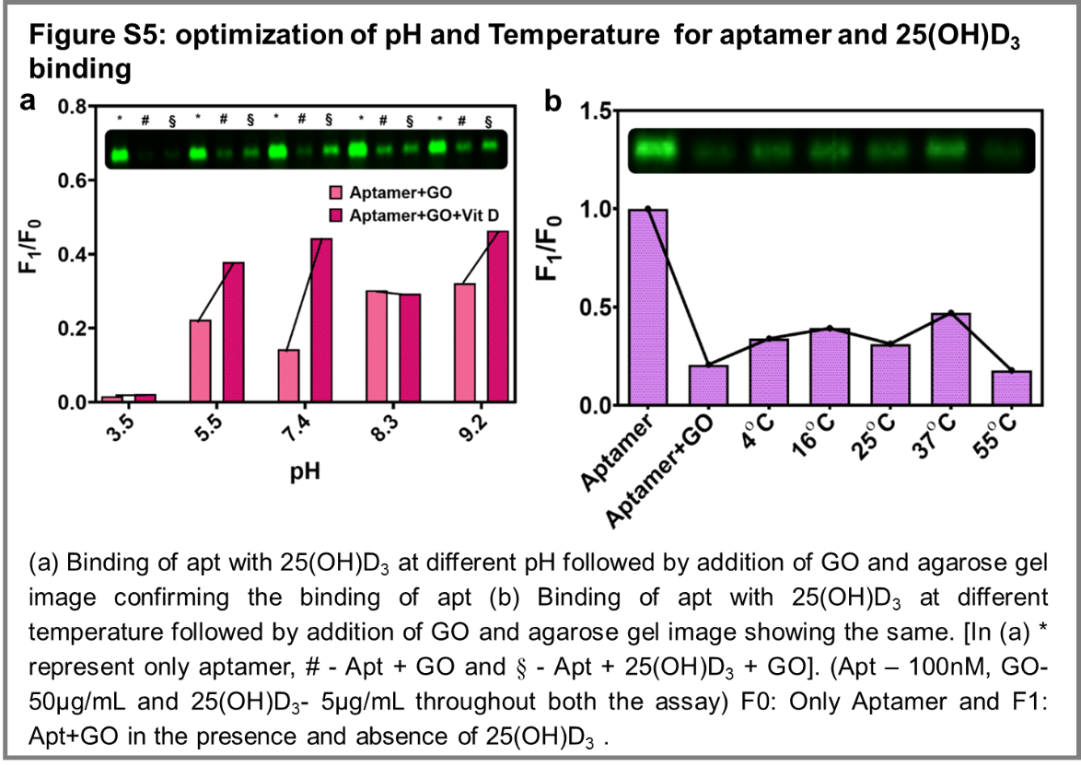
**

*6. Specificity analysis with scrambled aptamers*

*
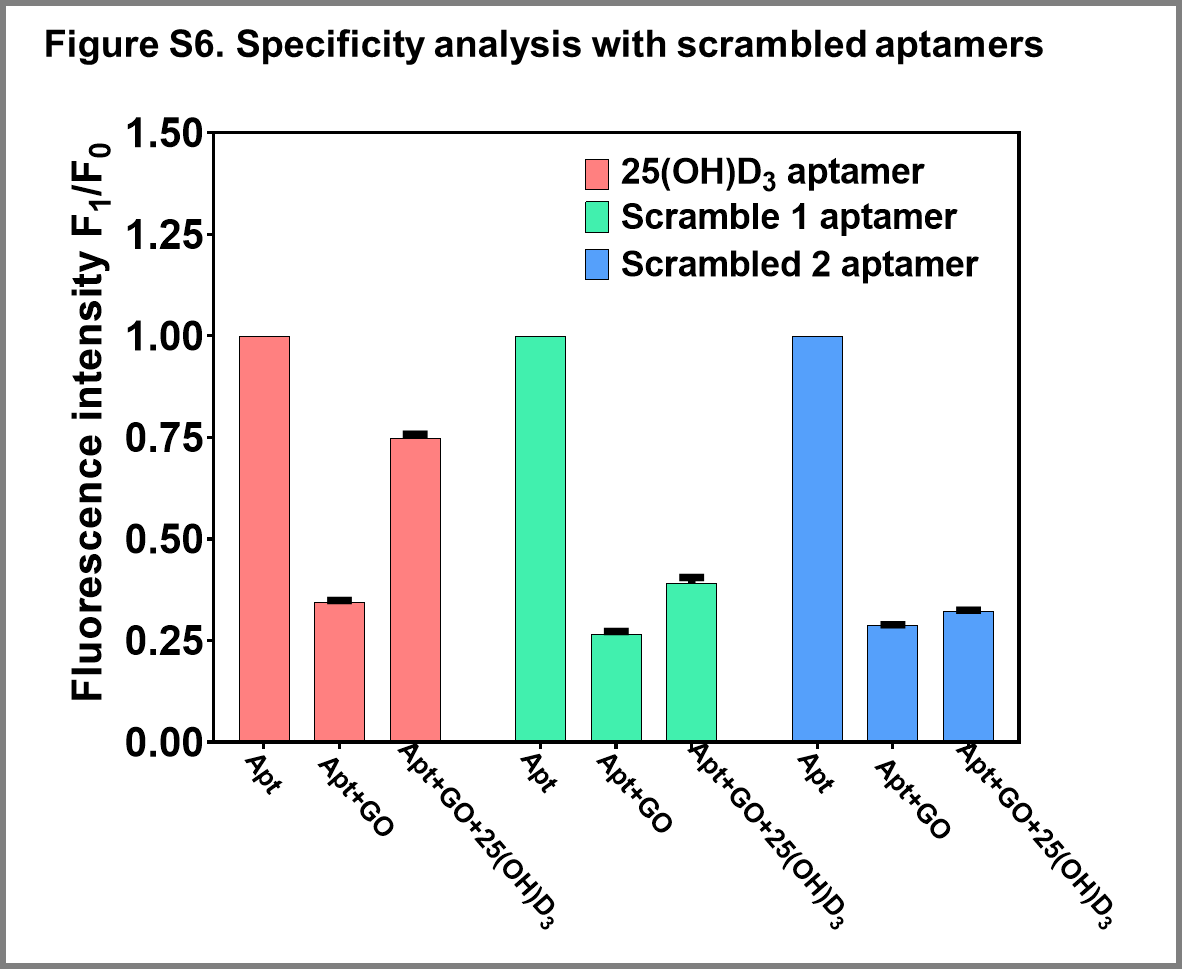
*

*7. Whole gel images:*

*
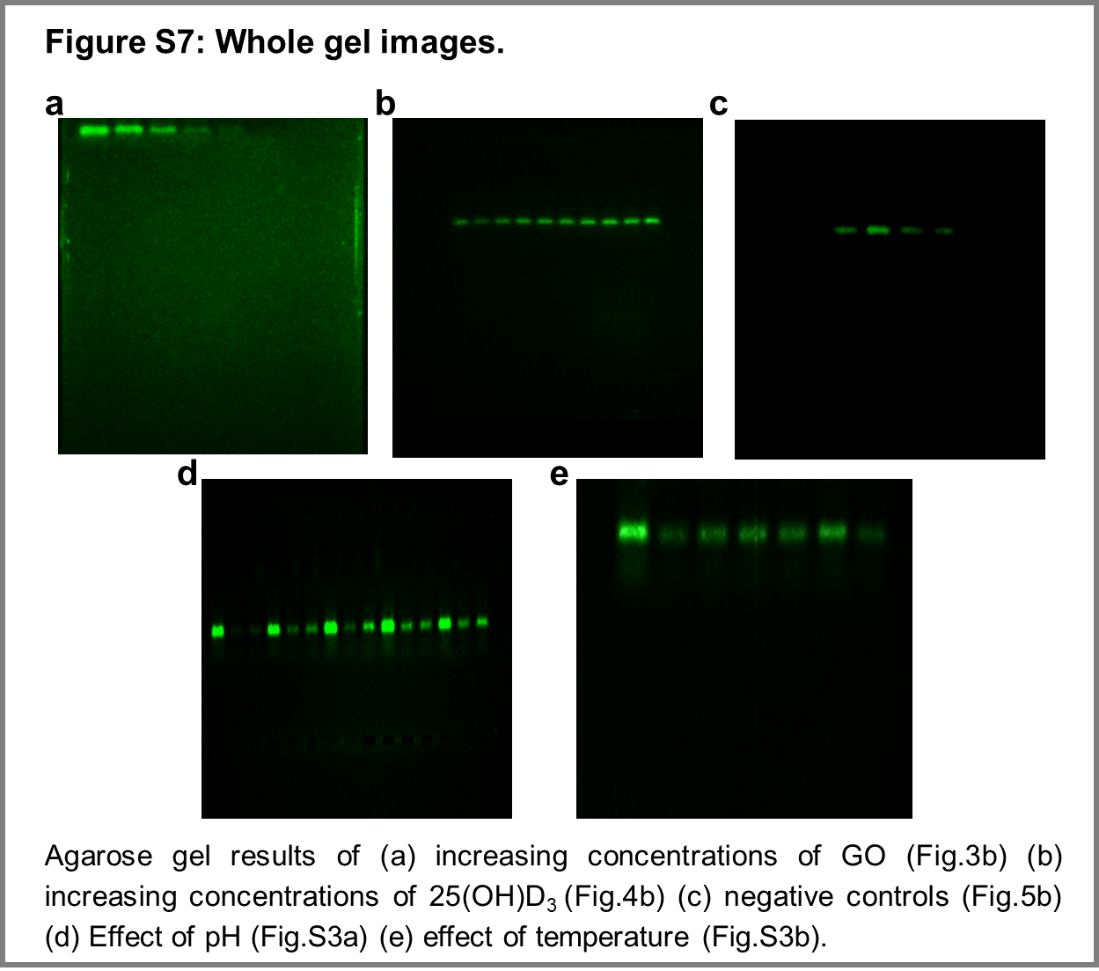
*

*8. Technical datasheet of aptamer*


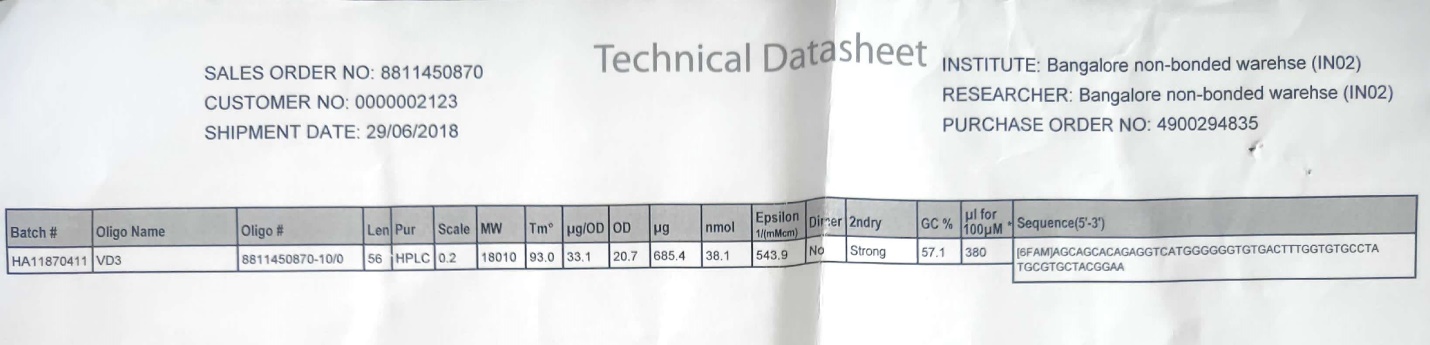

Supplement: Supplementary file 1 — Supplementary Information. [file 41598_2021_2837_MOESM1_ESM.docx]
